# A Novel Tetraenoic Fatty Acid Isolated from *Amaranthus spinosus* Inhibits Proliferation and Induces Apoptosis of Human Liver Cancer Cells

**DOI:** 10.3390/ijms17101604

**Published:** 2016-09-22

**Authors:** Arijit Mondal, Tanmoy Guria, Tapan Kumar Maity, Anupam Bishayee

**Affiliations:** 1 Department of Pharmaceutical Chemistry, Bengal College of Pharmaceutical Sciences and Research, Durgapur 713 212, West Bengal, India; 2 Department of Pharmaceutical Technology, Jadavpur University, Kolkata 700 032, West Bengal, India; tguria.ju@gmail.com (T.G.); jutkmaity@yahoo.com (T.K.M.); 3 Department of Pharmaceutical Sciences, College of Pharmacy, Larkin Health Sciences Institute, Miami, FL 33169, USA

**Keywords:** *Amaranthus spinosus*, fatty acid, hepatocellular carcinoma, antiproliferative effect, apoptosis, cell cycle regulation

## Abstract

*Amaranthus spinosus* Linn. (Family: Amaranthaceae) has been shown to be useful in preventing and mitigating adverse pathophysiological conditions and complex diseases. However, only limited information is available on the anticancer potential of this plant. In this study, we examined the antiproliferative and pro-apoptotic effects of a novel fatty acid isolated from *A. spinosus*—(14*E*,18*E*,22*E*,26*E*)-methyl nonacosa-14,18,22,26 tetraenoate—against HepG2 human liver cancer cells. We used 3-(4,5-dimethylthiazol-2-yl)-2,5-diphenyltetrazolium bromide (MTT) assay to determine cell viability, flow cytometry assay for cell cycle analysis, and Western blot analysis to measure protein expression of Cdc2), cyclin B1, Bcl-2-associated X protein (Bax), and B-cell lymphoma 2 (Bcl-2). The MTT assay showed that the fatty acid markedly inhibited the proliferation of HepG2 cells in a dosage-dependent fashion, with a half maximal inhibitory concentration (IC_50_) value of 25.52 µmol/L. This antiproliferative result was superior to that of another known fatty acid, linoleic acid (IC_50_ 38.65 µmol/L), but comparable to that of standard anticancer drug doxorubicin (IC_50_ 24.68 µmol/L). The novel fatty acid also induced apoptosis mediated by downregulation of cyclin B1, upregulation of Bax, and downregulation of Bcl-2, resulting in the G_2_/M transition arrest. Our results provide the first experimental evidence that a novel fatty acid isolated from *A. spinosus* exhibits significant antiproliferative activity mediated through the induction of apoptosis in HepG2 cells. These encouraging results may facilitate the development of *A. spinosus* fatty acid for the prevention and intervention of hepatocellular carcinoma.

## 1. Introduction

Cancer has become an enormous global health burden, because there are approximately 12.7 million cancer cases and 7.6 million cancer deaths every year [1,2]. Chemotherapy has been considered as the most efficient method for cancer therapy, but most cancer patients suffer from toxicity and side effects associated with chemotherapy.

Hepatocellular carcinoma (HCC), the most predominant pattern of liver cancer, is the sixth most common cancer and the third leading cause of cancer mortality worldwide [3]. The incidence of HCC in the United States has dramatically increased by more than 70% in the past 25 years [4]. According to the American Cancer Society, an estimated 35,660 new instances of liver cancer (including intrahepatic bile duct cancers) are anticipated to take place in the United States during 2015, approximately three-quarters of which are expected to be HCC. Liver cancer incidence rates are around three times higher in adult males than in women, and have doubled in each sex over the past two decades [5]. The majority of HCC cases are attributable to underlying infections caused by hepatitis B and C viruses [6]. In India, 70% to 80% of all HCCs are related to the hepatitis B virus (HBV), about 15% are linked to hepatitis C virus (HCV), and 5% to both HBV and HCV. Incidence of HCC in India occurs in individuals between 40 to 55 years, and also above 60 years. Eighty percent of all HCCs in India occur with liver cirrhosis in the background, and 60% of all these cases are hepatitis B-positive carriers [7]. Moreover, several other risk factors, such as alcohol consumption, obesity, iron overload, environmental pollutants, and dietary carcinogens (i.e., aflatoxins and nitrosamines) have been proven to play active roles in HCC etiology [8]. Still, no proven effective systemic chemotherapy is available for HCC patients.

Considering the lack of effective treatment and the grave prognosis of HCC, chemoprevention (use of natural and synthetic agents to reverse, suppress, or prevent carcinogenesis) has been considered to be the best strategy in reversing the current morbidity and mortality associated with this disease [9]. Emerging studies provide compelling evidence that various phytoconstituents, derived from dietary and medicinal plants, exhibit significant promise in the prevention and management of HCC [10].

**Figure 1:**
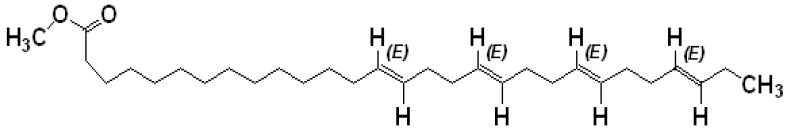
Structure of the fatty acid isolated from the chloroform fraction of *Amaranthus spinosus*.

*Amaranthus spinosus* Linn. (Family: Amaranthaceae), commonly known as “spiny pigweed” or “spiny amaranth”, is used as vegetable and cultivated throughout India, Sri Lanka, and many tropical countries [11]. Traditionally, this plant has been used in diabetic complications, as a diuretic, analgesic, antipyretic, antileprotic agent, and in the treatment of bronchitis and piles [12]. *A. spinosus* is reported to possess antidepressant [13], anti-inflammatory [14], antimalarial [15], antiandrogenic [16], antihyperlipidemic, and spermatogenic activities [17]. Administration of *A. spinosus* extract restored the altered hematological parameters in pigs [18] and normalized biochemical changes in the epididymis of rats [19]. Amaranthine, isoamaranthine, hydroxycinnamates, rutin, quercetin, and kaempferol glycosides are some of the phytochemical constituents identified in *A. spinosus*. According to previous studies, leaves of *A. spinosus* possess significant antitumor and cytotoxic activities [20,21]. However, the active constituents responsible for such activities were not specified. In this respect, we have recently isolated and purified one novel fatty acid—namely,(14*E*,18*E*,22*E*,26*E*)-methyl nonacosa-14,18,22,26 tetraenoate (Figure 1)—from the whole plant of *A. spinosus,* and reported antidiabetic activity of this fatty acid [22]. Nevertheless, the potential anticancer effect of the aforementioned fatty acid has yet to be examined, to our knowledge. Thus, in the present study, we have investigated the effect of the previously isolated fatty acid against the growth of HepG2 human liver cancer cells and defined the underlying mechanisms of action.

## 2. Results

### 2.1. The Purified Fatty Acid Exerts an Antiproliferative Effect

**Figure 2:**
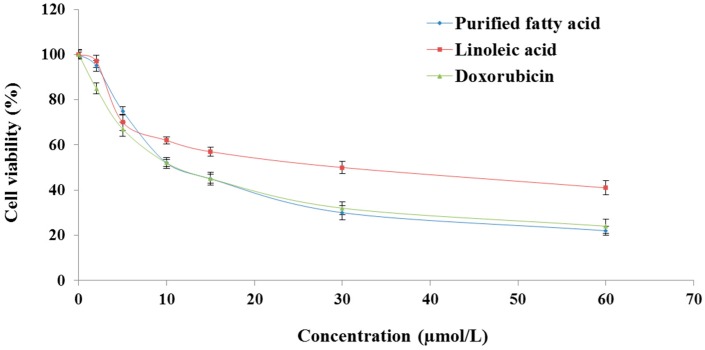
In vitro cytotoxic effects of purified fatty acid from *A. spinosus*, linoleic acid, and doxorubicin in HepG2 cells. HepG2 cells were seeded in a 96-well plate (10,000 cells/well) 24 h prior to the addition of each test material. Following 48h incubation with the extract, cell proliferation was determined by the 3-[4,5-dimethylthiazol-2-yl]-2,5-diphenyltetrazolium (MTT) assay. Each data point represents mean ± standard deviation based on quadruplicate determinations in three to five independent experiments.

3-[4,5-Dimethylthiazol-2-yl]-2,5-diphenyltetrazolium (MTT) assay indicated that the fatty acid isolated from *A. spinosus* markedly inhibited the proliferation of HepG2 cells in a dosage-dependent fashion (Figure 2), and the half maximal inhibitory concentration (IC_50_) value was found to be 25.52 µmol/L (Figure 2). On the otherhand, linoleic acid (another fatty acid) and doxorubicin (a standard anticancer drug) showed growth inhibitory effects in HepG2 cells (Figure 2), with IC_50_ values of 38.65 and 24.68 µmol/L, respectively. This outcome reflects a promising anticancer activity of the purified fatty acid against HCC comparable to doxorubicin, but superior to linoleic acid.

### 2.2. The Purified Fatty Acid Induces Apoptosis

**Figure 3:**
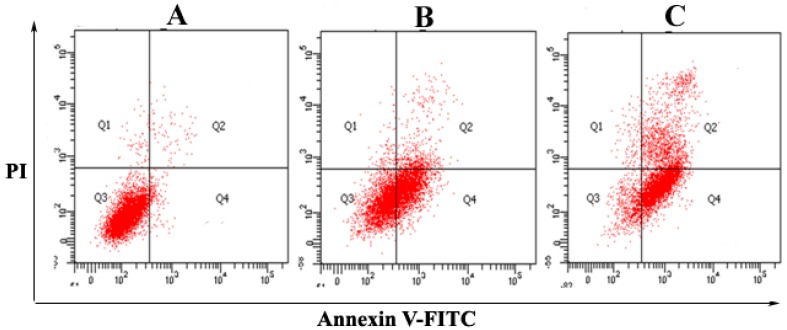
Flow cytometric analyses of *A. spinosus* fatty acid-induced apoptosis in HepG2 cells using annexin V-fluorescein isothiocyanate (FITC)/propidium iodide (PI). Quadrants: Q3 (normal cells), Q4 (apoptotic cells), Q2 (late apoptotic/necrotic cells). The mean apoptotic population for (**A**) control (normal cells); (**B**) 25 µmol/L; and (**C**) 60 µmol/L of fatty acid-treated HepG2 cells in 24 h, respectively.

To confirm that the fatty acid induces cellular apoptosis, the proportion of cells in sub-G_1_ phase was evaluated. As depicted in Figure 3, the dual parameter fluorescent dot plot shows the viable cell population in the Q3 quadrant (annexin V−/propidium iodide (PI)−), the cells in early apoptosis in the Q4 quadrant (annexin V+/PI−), and the cells at the late apoptotic/necrotic stage in the Q2 quadrant (annexin V+/PI+). Figure 3A shows the mean apoptotic population (annexin V+/PI−) of HepG2 cells, which was 2.5%, 47%, and 68% for the control, 25 and 60 µmol/L of the fatty acid-treated HepG2 cells in 24 h, respectively. The results strongly indicated that the fatty acid possessed substantial apoptosis-inducing effect on the cancer cells. As portrayed in Figure 3A, in untreated cells, 2.5% of the cells were annexin V+/PI−, whereas 1.1% of the cells were annexin V+/PI+ (double positive). After treatment with 25 and 60 µmol/L of the fatty acid for 24 h, the double positive cell populations were found to be 4% (Figure 3B) and 28.4% (Figure 3C), respectively.

### 2.3. Purified Fatty Acid Exhibits Cell Cycle Arrest

**Figure 4:**
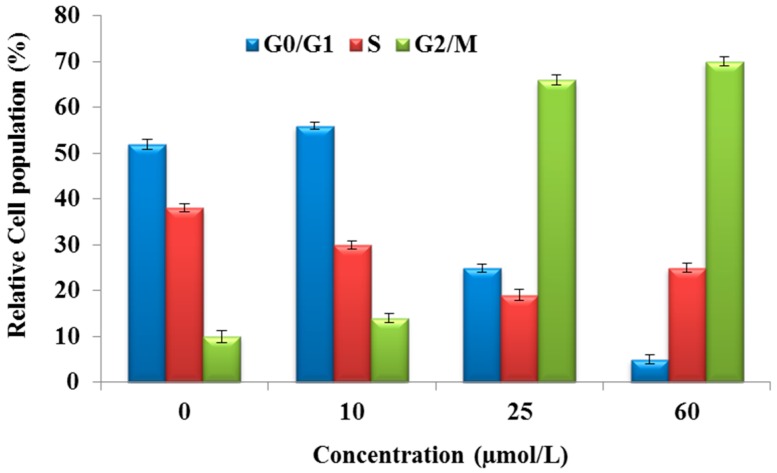
Cell cycle distribution of HepG2 cells with or without treatment with *A. spinosus* fatty acid at various concentrations after 24 h. The percentage of cells in each phase was estimated by flow cytometry.

The cell cycle consists of four distinct phases, which are cell growth (G_1_ phase), DNA synthesis (S phase), cell division (G_2_ phase), and mitosis and DNA replication (M phase). The alteration in cell cycle induced by the fatty acid was assessed by flow cytometry using PI staining of HepG2 cells after 24 h. It was found that the mean percentage of cells in the G_2_ phase increased from 10% ± 1.31% to 14% ± 1.06%, 66% ± 1.03%, and 70% ± 0.99% after treatment with 0, 10, 25, and 60 µmol/L fatty acid, respectively (Figure 4). Our results indicated that the fatty acid treatment could arrest cells in the G_2_/M phases, which might be ascribable to the destruction of DNA replication and mitosis processes of HepG2 cells. This growth was accompanied by a reduction in the number of G_0_/G_1_ phase cells.

### 2.4. Purified Fatty Acid Alters the Expression of Apoptosis-Associated Proteins

**Figure 5:**
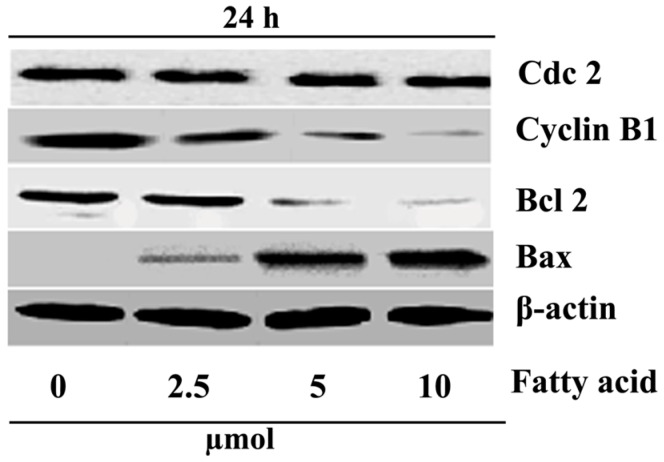
Western blot analysis of expression of proteins involved in apoptosis and G_2_/M arrest of HepG2 cells exposed to *A. spinosus* fatty acid. HepG2 cells were treated with several concentrations (0–10 µmol/L) of fatty acid for 24 h, which altered the expression of various proteins.

The expression levels of several proteins related to the cell cycle in HepG2 cells treated with *A. spinosus* fatty acid were evaluated using Western blot analyses. It is known that cell division cycle protein 2 homolog (Cdc2) and cyclin B1 have a close relationship with G_2_/M arrest. B-cell lymphoma 2 (Bcl-2) is an anti-apoptotic molecule, and Bcl-2-associated X protein (Bax) promotes apoptosis. HepG2 cells were treated with the fatty acid at different concentrations (0, 10, 25, and 60 µmol/L) for 24 h. β-Actin was used as internal control. As depicted in Figure 5, the level of Cdc2 was not altered, the expressions of cyclin B1 and Bcl-2 were decreased, and the expression of Bax increased after treatment with the fatty acid. *A. spinosus* fatty acid registered these results in a dosage-dependent fashion.

## 3. Discussion

Bioactive plant extracts are becoming useful resources to develop less toxic and more effective drugs to manage cancer progression. Cytotoxicity and antitumor effects of different extracts from *A. spinosus* have recently been reported [20,21], but the active constituents responsible for such activities have not been identified. Accordingly, the present work was initiated to probe into a possible anticarcinogenic effect and the associated cellular and molecular mechanisms of action of a novel fatty acid isolated from the chloroform fraction of *A. spinosus*.

The isolated and purified fatty acid from *A. spinosus* was found to be involved in inhibiting the proliferation of HepG2 cells, based on results from the MTT cytotoxicity assay. The fatty acid-induced apoptosis in HepG2 cells was confirmed by annexin V/fluorescein isothiocyanate (FITC)/PI staining utilizing a flow cytometric technique. Our results showed that the fatty acid induced significant cell cycle alteration and arrested cancer cells at the G_0_/G_1_ interface, which indicates the antiproliferative potency of the *A. spinosus* fatty acid.

It is well known that cell cycle dysregulation is a characteristic of tumor cells. To investigate a useful anticancer target, it is essential to study the proteins that actually mediate the critical events of the cell cycle. During G_2_/M transition, the cyclin-dependent kinases (CDKs) are activated, which, in turn, mediate the cell cycle after binding to a specific regulatory subunit, called cyclin. Together, these cyclin/CDK complexes are known to be a cell cycle regulator. The B-type cyclins, together with Cdc2, control the mitotic entry process. Without the synthesis of cyclin B prior to the G_2_/M transition, Cdc2 is not activated, and subsequently, cells cannot enter mitosis. Thus, the cell cycle will arrest at the G_2_ phase. Accordingly, these complexes (cyclin B/Cdc2) are called the M phase-promoting factor. Eventually, degradation of cyclin B by ubiquitin is the essential step for cells to exit mitosis. Our results indicated that the fatty acid could arrest HepG2 cells in the G_2_/M phase, suggesting that the fatty acid might contribute some effect to the dissociation of the cyclin B1/Cdc2 complex. The Western blot analysis exhibited that the expression of cyclin B1 was decreased, but that of Cdc2 remained unaffected.

Overall results suggested that the fatty acid was capable of arresting the cells at the G_2_ phase and preventing them from entering the mitosis process. Generally, apoptosis is regulated biologically via two major pathways: the extrinsic pathway and the intrinsic pathway [19]. The intrinsic mitochondrial pathway is controlled by Bcl-2 family proteins. In humans, more than 20 members of this family have been identified so far, including proteins that suppress apoptosis (Bcl-2, Bcl-xL, Mcl-1 and A1), and proteins that promote apoptosis (e.g., Bax, Bak, Bad and Bid) [23]. The pro-apoptotic and anti-apoptotic proteins of the Bcl-2 family may turn on and off apoptosis because of the formation of heterodimers among these proteins [24,25,26]. The heterodimerization results in mutual neutralization of the bound pro- and anti-apoptotic proteins. For this reason, anti-apoptotic Bcl-2 family proteins are important players in cancer cell survival and are recognized as relevant targets in cancer treatment. In HepG2 cells treated with the tested fatty acid, the level of Bax was increased, and concomitantly that of Bcl-2 was decreased, suggesting that the fatty acid induced apoptosis through upregulation of Bax and downregulation of Bcl-2.

## 4. Materials and Methods

### 4.1. Chemicals and Reagents

Linoleic acid was procured from Hi-Media Laboratories Pvt. Ltd. (Mumbai, Maharastra, India), and doxorubicin was obtained from Biochem Pharmaceutical Industries (Mumbai, Maharastra, India). Dulbecco’s modified Eagle medium (DMEM), l-glutamine, fetal bovine serum (FBS), penicillin–streptomycin, and trypsin-ethylenediaminetetraacetic acid (EDTA) were obtained from Gibco BRL (Grand Island, NY, USA). MTT azocasein, sodium dodecyl sulfate (SDS), Tris, glycine, β-mercaptoethanol (β-ME), CoomassieBrilliant Blue R-250, acrylamide/bisacrylamide, Brij-35 solution, propidium iodide (PI), Triton X-100, and ribonucleaseA were obtained from Sigma-Aldrich Chemical Co. (St. Louis, MO, USA). Dimethyl sulfoxide (DMSO) and Tris-EDTA (TE) buffer were purchased from Merck Co. (Darmstadt, Germany). Antibodies to Cdc2, cyclin B1, Bax, Bcl-2, and β-actin, as well as horseradish peroxidase (HRP)-conjugated secondary antibodies were obtained from Santa Cruz Biotechnology (Santa Cruz, CA, USA). All other chemicals and reagents (analytical grade) were purchased from Merck (Bangalore, Karnataka, India) and Thermo Fisher Scientific International, Inc. (Mumbai, Maharastra, India).

### 4.2. Plant Material, Extraction, and Isolation

The whole plant was collected in the month of June 2011, and was identified by Dr. V.P. Prasad, Scientist “D”, Botanical Survey of India, Howrah, West Bengal, India. A voucher specimen (No. CNH/18/2011/Tech.II/419) was deposited in the Department of Pharmaceutical Technology, Jadavpur University, Kolkata, West Bengal, India. The freshly collected plant material was washed, shade-dried, and then milled to coarse powder by a mechanical grinder for further studies.

The powdered plant material (2 kg) was extracted with methanol at room temperature for 48 h. The extract was separated out and dried under reduced pressure at 45 °C to afford a crude methanol extract (59.46 g). A part of the methanol extract (50 g) was suspended in water and fractioned successively with chloroform, ethyl acetate, and *n*-hexane. Then, the extracts were evaporated under reduced pressure in a rotatory evaporator and lyophilized to give remainders of the chloroform (7.76 g), ethyl acetate (7.06 g), and *n*-hexane (20.12 g) fractions. All the fractions were evaluated for anticancer activity. The chloroform fraction was found to be more potent than the ethyl acetate and *n*-hexane fractions. Hence, the chloroform fraction was further exploited, which contributed to the isolation of a novel fatty acid. The isolated compound was characterized as (14*E*,18*E*,22*E*,26*E*)-methyl nonacosa-14,18,22,26 tetraenoate, based on spectrographic analysis (infrared, proton nuclear magnetic resonance, and mass spectrometry). The detailed isolation procedure and structural elucidation of this fatty acid were reported in our previous publication [22].

### 4.3. Cell Culture

The HepG2 human liver carcinoma cell line was purchased from the National Centre for Cell Science (Pune, Maharastra, India). Cells were grown and maintained in DMEM supplemented with 10% fetal bovine serum, 100 µg/mL of penicillin, and 100 µg/mL of streptomycin. Cells were maintained at 37 °C in a humidified atmosphere of 5% CO_2_ in air. When the cells were 60%–70% confluent, the medium was aspirated, the cells were washed with phosphate-buffered saline (PBS), and fresh DMEM with or without antibiotic was added. Control plates were replenished with fresh medium and also incubated at similar conditions, as stated above.

### 4.4. Cell Viability Determination

Firstly, the MTT assay was performed to evaluate the antiproliferative effect of various test materials. HepG2 cells were seeded in a 96-well plate at a density of 1.0 × 10^4^ cells/well [27]. For the untreated cells (control), vehicle DMSO was added instead of the sample or test drug. The novel fatty acid from *A. spinosus*, linoleic acid, or doxorubicin at various concentrations (0–60 µmol/L) were added to each well separately and cultured for 48 h, followed by incubation with 5 mg/mL MTT for 4 h at 37°C. The culture medium was removed after centrifugation and replaced with 150 mL DMSO to dissolve the formazan crystals. The amount of formazan product was evaluated at 490 nm and 570 nm using a microplate reader. The cell viability was assessed as follows:

% cell viability = [absorbance of treated cells/absorbance of untreated cells] × 100

Subsequently, the IC_50_ value was calculated, setting the viability of untreated cells as 100%.

### 4.5. Cell Cycle Arrest Analysis

HepG2 cells (1 × 10^6^) were fixed with ice-cold 70% ethanol at 4 °C overnight, washed twice with PBS, then incubated in RNase A/PBS (100 mg/mL) at 37 °C for 30 min. DNA was labeled with PI (50 mg/mL), and the fluorescence was measured by a fluorescence-activated cell sorting (FACS) instrument (LSR Fortessa, BD Biosciences, Sparks, MD, USA). Data accumulation and analysis of the cell cycle distribution were performed using FACS Diva software (BD Biosciences) [28].

### 4.6. Apoptosis Assay by Annexin V-FITC/PI

Surface exposure of phosphatidylserine in apoptotic cells were quantitatively detected using annexin V-FITC/PI apoptosis detection kit (BD Biosciences) following the manufacturer’s protocol. Cells were incubated with the purified fatty acid at the concentration of 60 µmol/L for 24 or 36 h. The adherent and floating cells were mixed and processed according to the manufacturer’s instructions and measured with FITC/PI flow cytometry to differentiate apoptotic cells (annexin-positive and PI-negative) from necrotic cells (annexin- and PI-positive) [23,29].

### 4.7. Western Blot Analysis

Cells (2 × 10^5^) were seeded in 3 cm culture dishes. After 24 h, the cells were incubated with or without fatty acid (at IC_50_) for ≈20 h. Cells were washed with ice-cold PBS and then lysed in lysis buffer (50 mM Tris-HCl (pH 7.4), 10 mM EDTA, 1% Triton-100, and 26% urea). Equal measures of protein (20 mg) in the cell extracts were fractionated by 10% sodium dodecyl sulfate-polyacrylamide gel electrophoresis (SDS-PAGE)and then transferred onto nitrocellulose membranes. The membranes were blocked in the 20 mM Tris-buffered saline–0.1% Tween (TBST) buffer containing 5% skim milk at 4 °C for 60 min and then probed with specific primary antibodies overnight at the same temperature. After washing with TBST for 1 h, the membranes were incubated with HRP-conjugated anti-rabbit/mouse IgG for 1 h at room temperature, and then washed with TBST for 1 h. Finally, immunoblot signals were determined using an enhanced chemiluminescence (ECL) Western blotting detection reagent (Thermo Fisher Scientific International, Inc.) [30].

### 4.8. Statistical Data Analysis

All statistical analyses were carried out using the Minitab (version16.1.0, Minitab Inc., Cubic Computing (P) Ltd., Bangalore, Karnataka, India). The results are shown as mean ± standard deviation for three to five independent experiments.

## 5. Conclusions

The results of our present investigation clearly indicate that a novel fatty acid from *A. spinosus* is a potent growth inhibitor of cultured HepG2 cancer cells. The growth inhibition is linked to the G_2_/M phase cell cycle arrest associated with the downregulation of cyclin B1. The fatty acid also displayed programmed cell death (apoptosis) associated with the upregulation of Bax and the downregulation of Bcl-2. These results provide the molecular basis for the observed anticancer effect of the fatty acid isolated from the dietary plant *A. spinosus*. Further research is underway on upstream factors leading to the decrease of cyclin B1 and subsequent cells arrest in the G_2_/M phase, as well as signaling pathways through which this fatty acid promotes HepG2 cells to undergo programmed cell death.
